# Double-Versus Single-Bundle Anterior Cruciate Ligament Reconstructive Surgery: A Prospective Study With >1 Year Follow-Up

**DOI:** 10.7759/cureus.42829

**Published:** 2023-08-01

**Authors:** Udayan Das, Gopabandhu Patra, Biswajit Das, Sandeep Pradhan

**Affiliations:** 1 Department of Orthopaedics, Kalinga Institute of Medical Sciences, Bhubaneswar, IND; 2 Department of Orthopaedics, Bhima Bhoi Medical College, Balangir, IND; 3 Department of Orthopaedics, Fakir Mohan Medical College, Balasore, IND

**Keywords:** double-bundle, postoperative, preoperative, acl tear, acl injury, single-bundle

## Abstract

Background

The increased prevalence of knee trauma predominantly adds to the anterior cruciate ligament (ACL) tear frequencies that require single- or double-bundle reconstructive surgeries. Few studies have demonstrated the superior results of double-bundle reconstruction compared to single-bundle approaches. This study investigated the knee function improvement capacity of both reconstruction techniques in patients with ACL tears.

Methods

Thirty cases with ACL tears have been enrolled and segregated equally in distinct (single-bundle versus double-bundle) batches. The diagnostic assessments were undertaken through comprehensive clinical history, knee radiographs, Lysholm scoring, the International Knee Documentation Committee (IKDC) scale, the Lachman analysis, the International Knee Documentation Committee (IKDC) scale, and the pivot shift method.

Results

After one year, there was a significant enhancement in the postoperative versus preoperative Lysholm scores in the single-bundle (58.5 ± 21.2 vs. 82.4 ± 26.2, p<0.001) and double-bundle (86.4 ± 22.8 vs 60.3 ± 19.2, p<0.001) groups. There was a significant improvement in the IKDC scores after a follow-up period of one year (p-value: 0.012 and p-value: 0.002, respectively) in both of the study batches. After a year of follow-up, Lysholm scores (p=0.352) and IKDC scores (p=0.574) between the study groups (82.4 ± 26.2 vs. 86.4 ± 22.8) were comparable.

Conclusion

The clinical outcomes remained comparable between subjects with single-bundle reconstruction versus double-bundle reconstruction subjects with ACL injuries. Findings were similar between the groups after one year and two years of surgical interventions.

## Introduction

The permanence of the knee joint is entirely based on anterior and posterior cruciate ligaments (or ACLs and PCLs) [1]. The high tensile strength of ACL is due to its collagen fibers and connective tissue bands that traverse across the intercondylar (anteromedial) region of the tibial plateau and femoral condyle [2]. The concomitant role of the crossed PCL and ACL is to hinder the excessive motion of the tibia (against the femur) beyond a limit in a backward/forward direction [3,4]. The knee joint’s rotational stability is primarily due to ACL’s capacity to resist valgus/varus stress [2]. Nearly 100,000-200,000 individuals in the United States experience knee injuries, which are predominantly based on ACL tears and sprains [1].

Sportspersons are highly prone to ACL injuries and often receive RICE therapy based on rest, application of ice, knee compression, and lower extremity elevation [1,5]. However, the nonoperative management of ACL injuries often leads to recurrent instabilities and adds to the risk of meniscal and chondral trauma [6]. The surgical repair of ACL utilizes suture anchors or sutures to reapproximate its torn extensions [7]. This surgical technique for ACL repair was initially developed in the early 1900s by Robson [8,9]. ACL repair begins with reconstructing the native ACL’s ruptured portion and subsequent reconfiguration of a new ligament via different grafts based on the quadriceps tendon, bone-patellar tendon-bone, and hamstring tendon [9]. The reconstructive approach aims to reestablish the native ACL anatomy by utilizing an allograft from a cadaver or an autograft from the patient’s body [10].

The high success rates of ligament reconstruction approaches are responsible for their frequent utilization for ACL injuries. Single-bundle reconstruction is currently the preferred approach for treating ACL injuries. Single-bundle reconstruction is widely practiced in open physes, particularly in patients with <12 mm notches and <14 mm tibial insertion sites. Evidence demonstrates the feasibility of ACL reconstruction for patients with concomitant Kellgren Lawrence changes (grade ≥3)/severe arthritis, severe bone bruising, and ligamentum injuries. Alternatively, the double-bundle reconstruction approach is usually practiced in closed physes, and in patients with intercondylar notch >14 mm and tibial insertion location >14 mm. The double-bundle reconstruction approach is, however, not recommended for patients with severe bone bruising, Kellgren Lawrence grade <3/advanced arthritis changes, and simultaneous ligament injuries. This procedure aims to reconstruct anteromedial and posterolateral bundles to effectively reproduce knee kinematics and native anatomy [9].

The outcomes from a few studies indicate the occurrence of residual knee instability after single-bundle reconstruction [11]. Evidence indicates the need for a supplementary graft during single-bundle reconstruction to improve knee stability [12]. The peroneus longus tendon (anterior half) and the semitendinosus supplementary grafts help reduce donor site morbidity and enhance knee strength and stability [13]. ACL repair via the double-bundle procedure is the modification of the single-bundle reconstruction approach that utilizes distinct grafts to replace the ACL bundles [14]. Real-world outcomes demonstrate the double-bundle ACL approach’s capacity in improving treatment results and knee kinematics, and normalizing the knee structure [15]. The evidence further reveals the synergistic role of posterolateral and anteromedial bundles in improving knee function [16]. They also enhance the motion of the knee joint ranges by improving its extension capacity and resistance to stress. Recent evidence indicates the role of the hamstring tendon based on the double-bundle approach in resuming the pre-injury activity of the traumatized knee [17].

Few studies demonstrate the superiority of double-bundle intervention to contemporary surgery in improving knee kinematics [18]. The single-bundle approach fails to completely improve the biomechanical motion range of the knee joint because of the restricted use of an anteromedial bundle for enhancing anterior stability [19]. Alternatively, a recent literature review and retrospective study reveals no significant differences in knee pain reduction and knee flexion/extension loss in patients who underwent single-bundle reconstruction [20]. However, these outcomes are of limited reliability due to their short-term follow-ups. A recent meta-analysis and systematic review provide contradictory evidence indicating improvements in functional outcomes and knee stability after double-bundle knee reconstruction compared with the contemporary approach [21]. These findings appear valid at the short-term follow-up; however, the long-term and mid-term follow-up outcomes reveal marked differences in knee function/stability after single- and double-bundle knee reestablishments. In addition, rare studies have compared the results of single- versus double-bundle anterior cruciate ligament tear surgeries in Indian populations. Accordingly, this single-center prospective study aims to associate the consequences of single-bundle and double-bundle reconstruction procedures in patients with ACL injuries.

## Materials and methods

This prospective randomized controlled study has been performed between October 2020 and July 2022 in the Department of Orthopedics, Kalinga Institute of Medical Sciences, Bhubaneswar (India). The study was performed after getting ethical committee approval (KIITS/0012/OR2018).

Thirty patients with a primary ACL injury who were admitted to the hospital for surgical management were included in the study. Patients with lateral or posterior cruciate ligament injuries were excluded from the study. Patients with a height <174 cm, a PCL-dominant intercondylar notch, a history of knee ligament surgery, arthritis, meniscal injury/meniscectomy, malignant changes in the knee joint(s), posterolateral knee instability, and knee fracture were also excluded from the study.

Two independent allocators randomly selected the enrolled patients for single- or double-bundle reconstructive surgery. Each of the study groups included 15 patients. After ethical approval of the study from the hospital, the enrolled patients provided written informed consent. The surgery procedures and possible outcomes/adversities were explained to all patients before the initiation of single- or double-bundle reconstructions. The preoperative and postoperative assessments included a comprehensive clinical history and standard knee radiographs (AP/lateral views) [22]. Other comprehensive knee assessments included the International Knee Documentation Committee (IKDC) scale, pivot shift test, Lachman test, and Lysholm score [23]. Postoperative observations were performed during the first month, third month, sixth month, and twelfth months, respectively. 

Surgical procedures

A group of four expert surgeons was randomized for undertaking the double-bundle and single-bundle ACL surgeries. Surgeons were shifted between the study groups and each surgeon performed ≥1 reconstruction/type. The routine preoperative arthroscopic examinations were undertaken after anesthesia to evaluate the ACL injury, meniscal tears, and joint stability. The anteromedial aspect of the knee joint was explored with the standard longitudinal incision. The semitendinosus/gracilis tendons were separated, and absorbable sutures were utilized to secure their friends. The harvesting of both tendons (20-22 cm) was performed, followed by reapproximating the Sartorius fascia to its insertion location. 

Single-Bundle ACL Reconstructive Surgery

**Figure 1:**
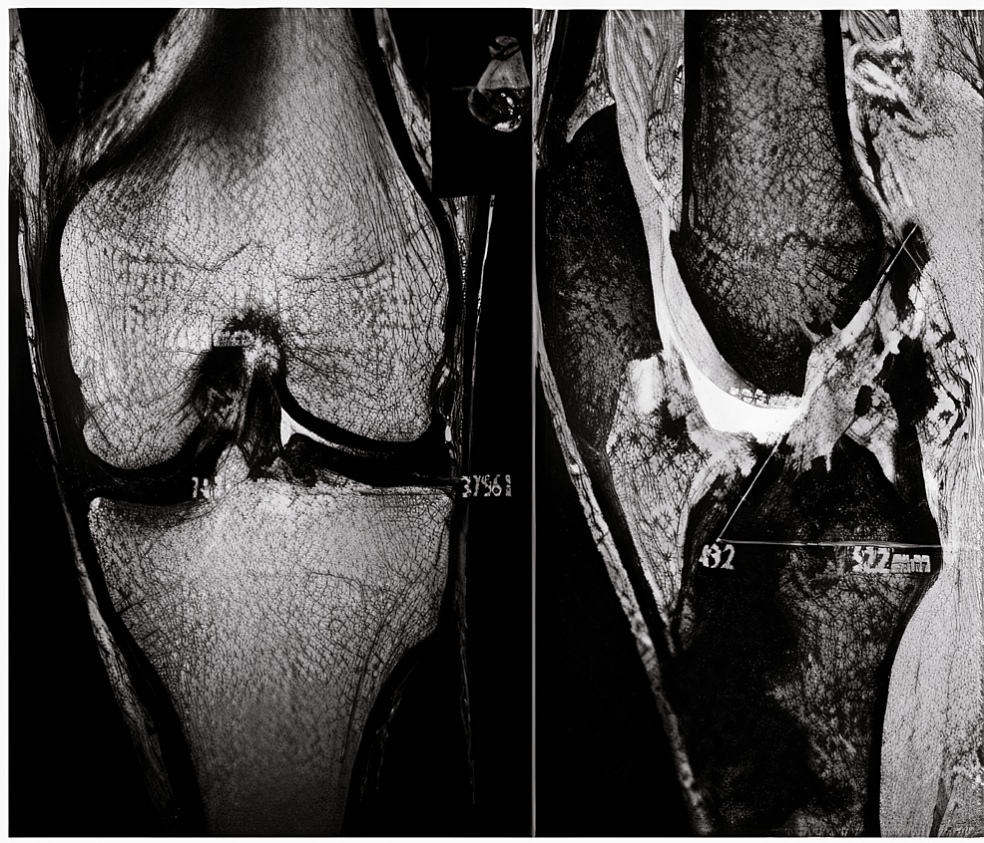
MRI showing the knee with single-bundle reconstruction

After securing the ACL stump, the posterior half of its footprint was utilized to construct a tibial tunnel. The measurement of the graft dimensions guided the preparation of the femoral tunnel (7-8 mm)/tibial tunnel (8-9 mm) diameters. A trans-portal approach was utilized for placing the femoral tunnel on the lateral femoral condyle. The operated knee was gradually bent while drilling the tunnel in the proximity of its extension threshold. Finally, at the knee flexion of 10-20°, ACL was secured across the femur (Figure 1). 

Double-Bundle Reconstruction of ACL

**Figure 2:**
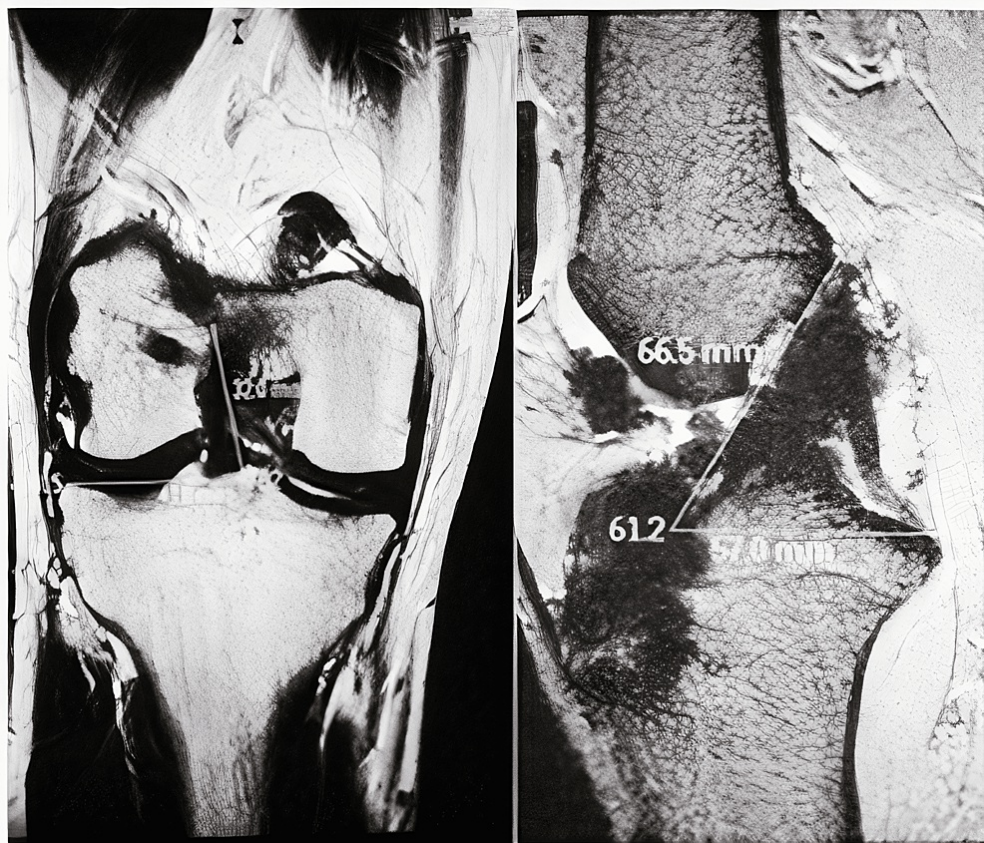
MRI showing the knee with double-bundle reconstruction

The semitendinosus and gracilis bundles were utilized to prepare the posterolateral and anteromedial bundles, respectively. The diameter of the graft guided the preparation of tunnels for both bundles. The length was measured to be over 7 cm, and the diameter was 7-9 mm. The graft was split into two bundles with the use of scissors (black arrow) along the natural plane of cleavage between the rectus femoris and vastus intermedius, providing the anteromedial and posterolateral bundles, which will enter the tibial tunnels during the double-bundle anterior cruciate ligament reconstruction. The anteromedial tunnel was constructed by the transtibial approach while the posterolateral femoral tunnel was created by femoral-aiming equipment following the outside-in technique. Finally, at the knee flexion of 60-70° and 10-20°, the posterolateral/anteromedial bundles were approximated to the femur and fixated on the tibia (Figure 2). These bundles were then fixed to the femur and tibia by the method of graft fixation while flexing the knee at 10-20° and 60-70°, respectively, using bioabsorbable screw fixation.

Statistical analysis

Microsoft Excel 2007 (Microsoft Corporation, Redmond, WA) was utilized for data collection by two independent data entry experts. SPSS software version 27 (IBM Corp., Armonk, NY) was used for the statistical analysis of the outcome variables. After the assessment of standard deviations/means of the quantitative variables, the paired t-test was done to associate the pre and post-operative outcomes. The reference (p<0.05) determined the statistical significance of these results. The continuous variables between the study groups were statistically compared by the independent samples t-test. In addition, the chi-squared test determined the association between the categorical variables.

## Results

Fifteen patients were placed in the single-bundle and 15 in the double-bundle groups. All 30 patients had a definitive diagnosis of ACL tear; the single-bundle and double-bundle groups had a mean age of 29.34 ± 5.42 years and 28.57 ± 4.57 years, respectively. No statistically significant difference occurred among both study groups' mean ages. The majority of study participants were males (11/15 in the single-bundle; 9/15 in the double-bundle); however, the gender difference between the groups lacked statistical significance (p=0.699). Table 1 depicts and compares the demographic characteristics (age and gender distribution) of patients in the single-bundle and double-bundle groups.

**Table 1:** Age and sex distribution between the groups DB: double-bundle; SB: single-bundle. NS: non-significant

Variables	SB Group (n= 15)	DB Group (n = 15)	P value
Age			
< 25 years	5 (33.3)	6 (40.0)	0.704 ^NS^
≥ 25 years	10 (66.7)	9 (60.0)	
Sex			
Male	11 (73.3)	9 (60.0)	0.699 ^NS^
Female	4 (26.7)	6 (40.0)	

The pre/postoperative Lysholm scores (after one year) in the single-bundle category were 58.5 ± 21.2 and 82.4 ± 26.2, respectively (p<0.001) (Table 2). Like the single-bundle group, a statistically relevant increase in the postoperative scores versus preoperative scores was also observed in the double-bundle group (86.4 ± 22.8 vs 60.3 ± 19.2, p<0.001). Statistically relevant improvements in IKDC scores were observed in both study groups after a one-year follow-up (p=0.012 and p=0.002) (Table 2).

**Table 2:** Pre- and postoperative follow-up comparisons of Lysholm and IKDC scores in both study groups DB: double-bundle; SB: single-bundle; IKDC: International Knee Documentation Committee * denotes statistically significant p<0.05

Parameters	SB Group			DB Group		
	Pre-op	One year follow-up	P value	Pre-op	One year follow-up	P value
Lysholm score (Mean ± SD )	58.5 ± 21.2	82.4 ± 26.2	<0.001*	60.3 ± 19.2	86.4 ± 22.8	<0.001*
IKDC score normal	0 (0)	0 (0)	0.012*	0 (0)	0 (0)	0.002*
Nearly normal	4 (26.7)	8 (53.3)		3 (20.0)	9 (60.0)	
Abnormal	8 (53.3)	7 (46.7)		8 (53.3)	6 (40.0)	
Severely abnormal	3 (20.0)	0 (0)		4 (26.7)	0 (0)	

**Figure 3:**
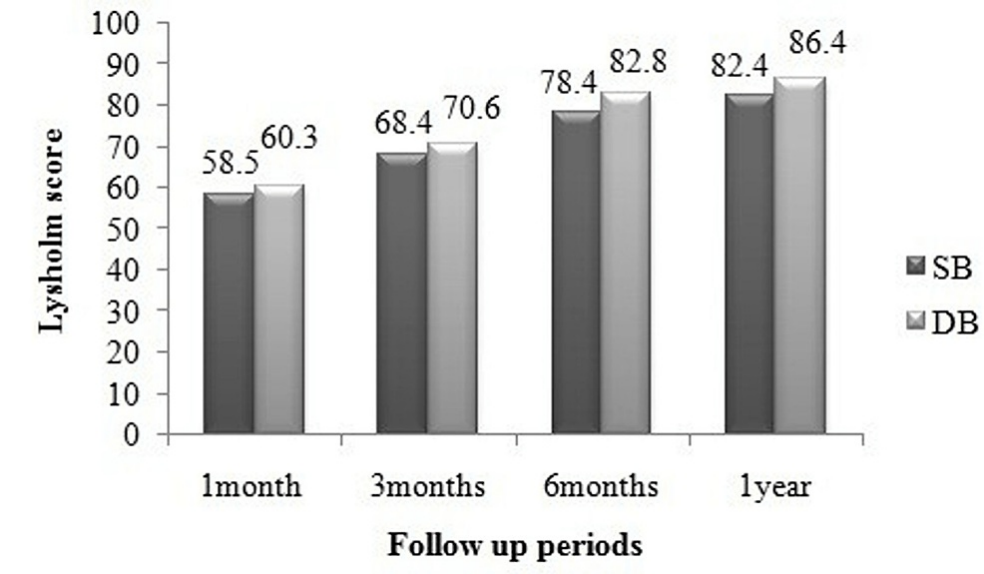
Lysholm score at different follow-up times in the single-bundle and double-bundle groups

A consistent increase in Lysholm scores was found at different follow-up points in both study groups. Figure 3 depicts significant variations in Lysholm scores in the single/double-bundle patients at one, three, six, and 12 months, respectively. Furthermore, after the one-year follow-up, no statistically significant differences were observed for the Lysholm (p=0.352) and IKDC (p=0.574) scales between the study groups (Table 3).

**Table 3:** Comparisons of the Lysholm and IKDC scores between the study groups after a one-year follow-up DB: double-bundle; SB: single-bundle; IKDC: International Knee Documentation Committee; NS: non-significant

	SB Group	DB Group	P value
Lyshom score (Mean, SD)	82.4 ± 26.2	86.4 ± 22.8	0.352 ^NS^
IKDC score			0.574 ^NS^
Normal	0 (0)	0 (0)	
Nearly normal	4 (26.7)	3 (20.0)	
Abnormal	8 (53.3)	8 (53.3)	
Severely abnormal	3 (20.0)	4 (26.7)	

## Discussion

This investigation revealed comparable outcomes following single/double-bundle interventions in patients with an ACL injury. Importantly, no meaningful difference in the outcomes from both surgical approaches was observed between the study groups. Both study groups showed considerable improvements in the Lysholm and IKDC scores following the one-year follow-up versus the preoperative findings. The lack of significant alterations in outcomes among the single- and double-bundle reconstruction surgeries indicated their comparative effectiveness in improving knee motion in patients with ACL injuries.

Single-bundle ACL repair is routinely undertaken following four to eight weeks of trauma, particularly when any joint swelling subsides and the injured knee attains the full range of motion [24]. The autografts are prepared with the gracilis and semitendinosus after harvesting the ipsilateral hamstring. A femoral tunnel is constructed to secure the hamstring graft, which is followed by femoral fixation. Alternatively, the double-bundle reconstruction restores the posterolateral and anteromedial ACL bundles to improve rotational and anteroposterior stability [25]. The double-bundle technique aims to restore the tension in both bundles by reconstructing ACL’s native anatomy. However, most studies to date provide no conclusive evidence concerning the differences in both techniques based on their capacity to enhance clinical outcomes, rotational stability, and anteroposterior laxity in patients with ACL injury [26]. The long-term implications of single- versus double reconstructions require further assessment by randomized controlled trials.

The analysis of the Swedish National Knee Ligament register revealed comparable low revision rates in patients with single- versus double-bundle reconstructive surgeries [27]. In addition, no statistically significant differences were recorded for EuroQol-5D (EQ-5D) and Osteoarthritis Outcome Scores (OOS) between the study groups. A meta-analysis with a five-year follow-up revealed no superiority of the double-bundle to the single-bundle procedure in terms of osteoarthritis changes, graft failure rate, clinical function, and knee stability [28]. However, the reliability of these outcomes was restricted to patients with autologous ACL reconstruction. A subgroup meta-assessment of >2 years of follow-up data revealed better rotational laxity and low anterior laxity in patients in patients with double-bundle versus single-bundle procedures [29]. However, this finding lacked validity due to potential limitations in the design of the included studies. Another meta-analysis by Meredick et al. revealed no meaningful differences in pivot-shift/KT-1000 arthrometer outcomes between patients with single-bundle and double-bundle reestablishments [30]. The outcomes did not signify the notion of better knee rotation after double-bundle vs single-bundle intervention in patients with ACL. The findings of this prospective study align with the current evidence that rejects the scope of attaining better/superior knee function after a double- versus single-bundle approach in patients with ACL.

The limitations of the study were as follows: the outcomes are restricted by its small sample size, prospective design, and limited follow-up duration. This study also did not evaluate and compare the quality-adjusted life years of patients prior to and following single- versus double-bundle reconstruction. These limitations restrict the generalizability of our results across wider patient populations.

## Conclusions

This study revealed no statistically significant differences in knee function in ACL patients with single-bundle vs double-bundle procedures. Findings were similar between the groups after one to two years of follow-ups. The comparable short- and long-term outcomes from both surgical techniques increase the scope of their utilization for ACL surgeries on a case-to-case basis. However, prospective studies should re-evaluate these outcomes with larger sample size and >5 years of follow-up duration to standardize single/double-bundle techniques for ACL repair and minimize the risk of potential complications.
